# Affective Circuitry Alterations in Patients with Trigeminal Neuralgia

**DOI:** 10.3389/fnana.2017.00073

**Published:** 2017-09-05

**Authors:** Dave J. Hayes, David Q. Chen, Jidan Zhong, Ariel Lin, Brendan Behan, Matthew Walker, Mojgan Hodaie

**Affiliations:** ^1^Psychology Department and Neuroscience Program, Union College Schenectady, NY, United States; ^2^Division of Brain, Imaging and Behaviour Systems Neuroscience and Division of Neurosurgery, Department of Surgery, Toronto Western Hospital, Krembil Research Institute, University Health Network, University of Toronto Toronto, ON, Canada

**Keywords:** DTI, dMRI, diffusion magnetic resonance, chronic neuropathic pain, gray matter, MRI, emotion, structural neuroimaging

## Abstract

Trigeminal neuralgia (TN) is a severe chronic neuropathic facial pain disorder. Affect-related behavioral and structural brain changes have been noted across chronic pain disorders, but have not been well-studied in TN. We examined the potential impact of TN (37 patients: 23 with right-sided TN, 14 with left-sided TN), compared to age- and sex-matched healthy controls, on three major white matter tracts responsible for carrying affect-related signals—i.e., cingulum, fornix, and medial forebrain bundle. Diffusion magnetic resonance imaging (dMRI), deterministic multi-tensor tractography for tract modeling, and a model-driven region-of-interest approach was used. We also used volumetric gray matter analysis on key targets of these pathways (i.e., hippocampus, cingulate cortex subregions, nucleus accumbens, and ventral diencephalon). Hypotheses included: (1) successful modeling of tracts; (2) altered white matter microstructure of the cingulum and medial forebrain bundle (via changes in dMRI metrics such as fractional anisotropy, and mean, axial, and radial diffusivities) compared to controls; (3) no alterations in the control region of the fornix; (4) corresponding decreases in gray matter volumes. Results showed (1) all 325 tracts were successfully modeled, although 11 were partially complete; (2) The cingulum and medial forebrain bundle (MFB) were altered in those with TN, with dMRI metric changes in the middle (*p* = 0.001) and posterior cingulum (*p* < 0.0001), and the MFB near the ventral tegmental area (MFB-VTA) (*p* = 0.001). The posterior cingulum and MFB-VTA also showed unilateral differences between right- and left-sided TN patients; (3) No differences were noted at any fornix subdivision; (4) decreased volumes were noted for the hippocampus, posterior cingulate, nucleus accumbens, and ventral diencephalon. Together, these results support the notion of selectively altered affective circuits in patients with TN, which may be related to the experience of negative affect and the increased comorbidity of mood and anxiety disorders in this population.

## Introduction

Trigeminal neuralgia (TN) is the most common chronic neuropathic facial pain disorder, characterized by the sudden onset of intermittent, intense, shock-like pain in distributions of the trigeminal nerve branches (Elias and Burchiel, 2002). Typically described as the worst pain imaginable, it is associated with high levels of negative affect and continued stress (Carlson, 2007; Vachon-presseau et al., 2013). Recently, diffusion magnetic resonance imaging (dMRI) was used to provide *in vivo* evidence of brain white matter alterations in TN patients, showing decreased fractional anisotropy (FA) and increased mean (MD), axial (AD), and radial (RD) diffusivities in affected trigeminal nerves (DeSouza et al., 2014). These dMRI metrics reflect the diffusivity of water along white matter tracts and support the presence of nerve alterations consistent with compression (Lutz et al., 2015). However, the neurovascular compression of the trigeminal nerve does not fully explain the disorder's presence, intensity, or clinical outcomes (Hilton et al., 1994; Miller et al., 2009; Hodaie and Coello, 2013; Ko et al., 2015). Moreover, changes in gray matter (GM) volumes and cortical thickness have been noted for clusters of voxels located within the anterior cingulate, thalamus, PAG, basal ganglia, insula, and orbitofrontal cortex (Desouza et al., 2013). Pharmacological treatments are not effective in many patients (Moulin et al., 2014) and, although neurosurgical treatments are capable of eliminating TN entirely in some cases, 20–30% of patients do not show improvement 1-year post-surgery (Hodaie and Coello, 2013). A better understanding of factors beyond nerve compression are needed to advance therapeutic decision making and improve prognostication. One promising avenue of inquiry focuses on classical affective or “limbic” circuitry as these are known to be involved in the regulation of states related to negative affect, pain, and stress. However, it is currently unclear if alterations in affective circuitry are related to the pathology of TN.

Human and animal studies have underscored altered psychological processing and affective circuitry in chronic neuropathic pain (Simons et al., 2013; Papini et al., 2015). For instance, an fMRI study showed that patients transitioning from acute to chronic back pain over a 1-year period had corresponding shifts in brain activity from classical pain-related (e.g., anterior cingulate cortex, anterior insula, thalamus) to more affect-related activations, involving the prefrontal cortex (PFC), amygdala, and nucleus accumbens/ventral striatum (NAc) (Hashmi et al., 2013; Vachon-Presseau et al., 2016). Patients with postherpetic neuralgia also showed altered affective circuit activations, including within the NAc and ventral tegmental area (VTA) (Geha et al., 2007), and those with fibromyalgia appear to show abnormal VTA responding to affective stimuli (Loggia et al., 2014). These are mirrored in animal studies which have identified changes in many cortical (e.g., cingulate, insula, hippocampus) and subcortical (e.g., NAc and VTA) regions (Yalcin et al., 2014). For instance, lesions of the rat cingulate cortex can reduce the aversiveness of neuropathic pain without impacting sensation, reward-related responding, or memory (Qu et al., 2011), while VTA disruptions caused by the upregulation of glial cells may be related to the persistence of chronic pain (Taylor et al., 2015).

Prior work from our group using a whole-brain, voxel-wise, analysis of white matter revealed lowered FA and higher MD in the middle cingulum, along with alterations in the corpus callosum, posterior corona radiata, and superior longitudinal fasciculus (DeSouza et al., 2014). Many neuroimaging studies have identified the cingulum in the processing of pain and negative affect (Hayes and Northoff, 2011, 2012; Shackman et al., 2011), and cingulotomy procedures in animals and humans can successfully reduce the unpleasantness of chronic pain (Yen et al., 2005; Qu et al., 2011). The use of whole-brain voxel-wise analytical programs has improved the objectivity of dMRI data at the group level, however, steps involving smoothing, registration, and masking significantly reduce the sensitivity of such analyses to uncover targeted abnormalities across subjects (for an in-depth discussion, including concerns related to white matter analysis using the widely-used software tract-based spatial statistics, see Bach et al., 2014). In the present work, we used an *a priori* neuroanatomical approach, which was highly sensitive to individual differences across subjects, to test the broad hypothesis that two major, well-defined, affective white matter tracts—the cingulum and medial forebrain bundle (MFB)—and their immediately targeted GM structures (i.e., cingulate cortex subregions, nucleus accumbens, and ventral diencephalon) would be altered in patients with TN compared to healthy controls.

Deterministic multi-tensor tractography was used to model three major affective white matter tracts (i.e., fornix, cingulum, MFB), followed by model-driven region-of-interest (ROI) analyses. The multi-tensor was chosen over a single-tensor approach as it has been shown to improve the modeling of tracts that are dense, highly crossing or angled, and travel over relatively long distances (Descoteaux et al., 2009; Qazi et al., 2009; Khalsa et al., 2014). The ROI approach avoided the problems that arise with the smoothing, registration, and masking required in many programs, such as tract-based spatial statistics, and allowed for a targeted subregional tract analysis at the individual level, in native space, which was analysable at the group level. With this combined approach, we tested the specific hypotheses that: (1) the fornix, cingulum, and MFB will be successfully modeled in TN patients, as well as healthy controls, using multi-tensor tractography; (2) ROI analysis from major subregions of the cingulum (i.e., anterior, middle, and posterior) and MFB (i.e., at the levels of the PFC, NAc, and VTA) will reveal differences in dMRI metrics (i.e., changes in FA, MD, AD, and RD) from controls consistent with the notion of altered affective circuitry in those with TN; (3) there will be no alterations in the fornix which will serve as a control region—as it has not been associated with chronic pain throughout the literature; (4) GM regions which are immediately innervated by the cingulum and MFB (i.e., cingulate cortex, nucleus accumbens, and ventral diencephalon) will show a decreased volume, reflecting the chronic impact of neuropathic pain.

## Materials and methods

### Participants

Retrospective analyses of dMRI data were carried out in 37 TN patients with unilateral pain (23 with right-sided, R-TN, and 14 with left-sided, L-TN, pain) and 28 healthy controls. All patients and volunteers were previously scanned at Toronto Western Hospital, and approval was granted by the University Health Network Research Ethics Board (Toronto, Canada). All healthy control volunteers gave written consent, while the UHN REB does not require this for retrospective analyses of patient data. Patient data was obtained through chart review, and the inclusion criteria were extreme unilateral pain consistent with a clinical diagnosis of TN, and not another disorder, as well as no prior brain surgeries. These patients were candidates for neurosurgery and, as such, were all treatment-resistant and reported the most intense subjective pain levels (e.g., a 9 or 10 on a 10-point scale) during clinical examinations. Patient demographic data can be seen in Table 1. R-TN (8 male; average age 47 ± 12; range 23–67 years) and L-TN (5 male; average age 55 ± 10; range 36–70 years) subjects were analyzed against sex- and age-matched healthy controls. The average ages and ranges for healthy controls of R-TN and L-TN patients, were 47 ± 12, range 23–65 and 53 ± 10, range 34–67, respectively. For optimal age- and sex- matching, 9 control subjects were used for both R-TN and L-TN analyses.

**Table 1:** Patient demographic data.

**Patient code**	**Side**	**Sex**	**Age (years)**	**Pain duration (years)**	**Medication (At time of surgery)**
1	L	M	67	7	Carbamazepine
2	L	M	49	0.5	Carbamazepine
3	L	F	65	7	Gabapentin, Lamotrigine, Oxcarbazepine
4	L	M	63	3	Duloxetine, Baclofen
5	L	F	70	–	None
6	L	F	53	8	Gabapentin
7	L	M	48	4	Carbamazepine
8	L	F	55	–	Pregabalin
9	L	F	50	2	Carbamazepine, Gabapentin
10	L	M	36	9	Carbamazepine
11	L	F	54	3	Carbamazepine, Ketorolac
12	L	F	42	3	Gabapentin, Nortriptyline
13	R	M	46	8	Ibuprofen
14	R	F	52	10	Duloxetine, Topiramate
15	R	M	43	2	Carbamazepine, Diazepam
16	R	F	61	4	Carbamazepine, Gabapentin
17	R	F	40	8	Baclofen, Gabapentin
18	R	F	33	5	Gabapentin
19	R	F	38	6	Carbamazepine
20	R	M	52	6	Carbamazepine, Hydromorphone
21	R	F	59	4	Carbamazepine, Pregabalin
22	R	F	38	5	Carbamazepine, Pregabalin
23	R	F	67	1	Gabapentin, Hydromorphone
24	R	F	63	7	Carbamazepine
25	L	F	64	2	Gabapentin
26	R	M	61	2	Pregabalin
27	L	F	49	–	Carbamazepine
28	R	F	45	4	None
29	R	F	47	2	Carbamazepine, Gabapentin
30	R	F	53	2	Carbamazepine
31	R	M	38	1	Carbamazepine, Pregabalin
32	R	M	23	–	Carbamazepine
33	R	F	52	–	None
34	R	F	52	5	Carbamazepine
35	R	M	24	–	Carbamazepine, Nortriptyline
36	R	F	63	12	Pregabalin
37	R	M	38	2	Carbamazepine

### MR acquisition and processing of diffusion-weighted and anatomical images

MR images were acquired on a GE Signa HDxt 3-tesla scanner (GE Healthcare, WI, USA) using an 8-channel phased-array head coil. Diffusion-weighted images were acquired over 12 min using 60 non-collinear directions with a dual-spin echo planar sequence to reduce eddy-current distortions. We used the Array Spatial Sensitivity Encoding Technique, or ASSET, with a factor of 2 and the following parameters: 0.94 × 0.94 × 3.0 mm voxels, 128 × 128 matrix, FOV = 24 cm, TE = 86.4 ms, TR = 12 s, *b* = 1,000 s/mm^2^. Images were processed using 3D Slicer v 4.3.1 (MA-MIC, http://www.slicer.org) (Pieper et al., 2004) and FSL v 5.0 (FMRIB Software Library, http://www.fmrib.ox.ac.uk/fsl/) (Smith et al., 2004) in a Linux environment. Diffusion-weighted scans were corrected for motion and eddy-current in FSL, and the *b-*matrices were corrected by averaging the rotational component of the gradient affine transforms and applying these to the original matrices (Leemans and Jones, 2009). 3D Slicer was used for visualization of diffusion magnetic resonance images (dMRI), seed and ROI drawing, tensor estimation, and the creation of scalar maps for fractional anisotropy (FA), and mean (MD), axial (AD), and radial (RD) diffusivities at the individual level. Anatomical T1-weighted axial images were acquired with a 3D Fast Spoiled Gradient Echo sequence, (0.9 × 0.9 × 1.0 mm^3^ voxels from a 256 × 256 matrix with a 5 ms echo time, 12 ms repetition time, and a 300 ms inversion time.

### Multi-tensor tractography modeling

**Figure 1:**
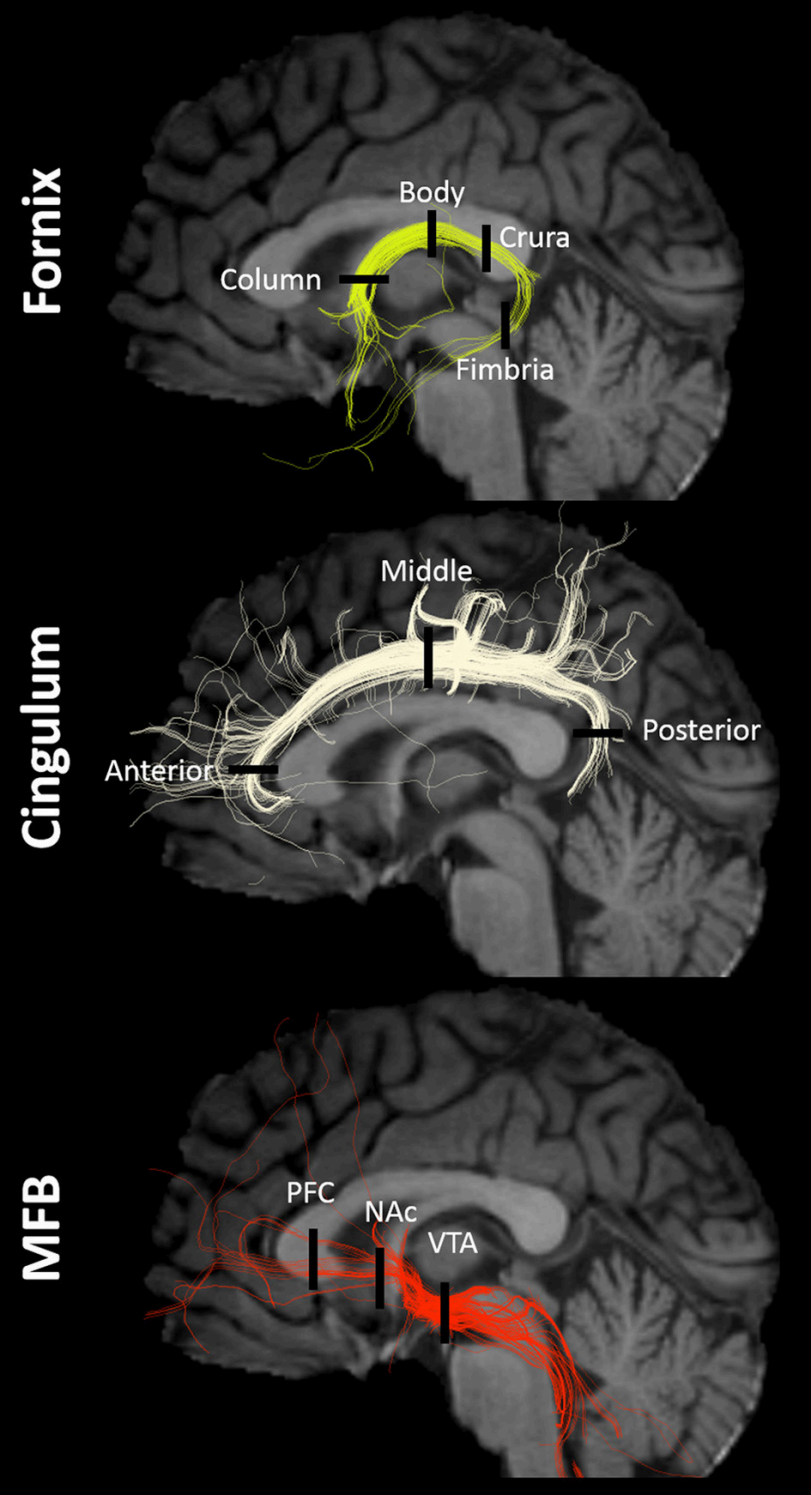
Tracts and ROIs. Example of fornix, cingulum, and MFB multi-tensor tractography models in a single subject with regions-of-interest (ROIs) indicated by black lines. NAc, nucleus accumbens/ventral striatum; PFC, prefrontal cortex; VTA, ventral tegmental area.

The fornix, and left and right cingula and MFB tracts were modeled from a single seed using the eXtended Streamline Tractography, or XST, deterministic multi-tensor tractographic algorithm (Qazi et al., 2009) (Figure 1). We chose a deterministic algorithm as Khalsa et al. (2014) showed that probabilistic is inferior to deterministic multi-tensor tractography in reconstructing longer brain pathways such as the ones described here (Khalsa et al., 2014). This resulted in the creation of 325 tractographic models across all subjects—65 midline forniceal models, 130 bilateral MFB, and 130 bilateral cingula models. Seeds to generate each tract were drawn in 3D Slicer using the FA map as a guide. Seed parameters were kept constant within tracts and across subjects: fornix seeded at body (height x width: 2 × 10 voxels in coronal plane; volume: 53 mm^3^), middle cingulum (2 × 8 voxels coronal; 42 mm^3^), MFB near VTA (2 × 5 voxels coronal; 26 mm^3^). The following multi-tensor tractography parameters were used: seeding point spacing of 0.25 mm, or 64 points/voxel, and streamline propagations initiated at fractional anisotropy > 0.2, tensor fractional = 0.1, curve radius = 0.8 radians, minimal path length = 10 mm, step size = 1 mm. In the few cases where the tract was incompletely modeled (see Results section below), we shifted the original seed in the most appropriate way (e.g., if the left fimbria was not complete, we shifted the original seed one voxel to the left) and remodeled until a complete tract was modeled (or could not be generated).

### Model-driven region-of-interest (ROI) white matter tract analysis

A ROI approach was used to investigate potential differences in dMRI metrics (FA, MD, AD, RD) across major anatomical subregions of the fornix, cingulum, and MFB (Figure 1). Six ROIs were drawn for each tract, resulting in 18 ROIs per subject, and placement was guided by the multi-tensor tractography model as described below. ROIs were drawn directly in native space (instead of using an inverse transformation from standard space) because using anatomical landmarks in conjunction with the tractography model allowed for the unbiased, individualized, placement of the ROIs. In other words, this model-driven approach allowed for a comparative analysis across patient and control groups based on highly individualized ROIs—similar to an approach that could be adopted in future for clinical use.

The inclusion criteria for ROI creation was as follows: (1) ROIs were anatomically bounded, meaning that the tractographic models and color-by-orientation FA maps were used to ensure that ROIs included voxels in biologically relevant areas (e.g., did not contain irrelevant/low FA voxels which could bias the diffusivity measures); (2) selected voxels were coextensive, meaning that only neighboring voxels around the points of highest density in the model were chosen; (3) ROIs were drawn in 2 adjacent slices (four slices for all fimbria ROIs in an attempt to account for the thinness of this tract); (4) selected voxels included at least three streamlines in the coronal plane or two streamlines in the axial plane, to account for the greater surface area of the coronal plane.

ROIs were chosen based on prior studies of the main anatomical divisions of the fornix (Chen et al., 2015), cingulum (Jones et al., 2013), and MFB (Coenen et al., 2011, 2012a). Descriptive anatomical localizations of each ROI were as follows (Figure 1). For the fornix: column (midline structure with vertical course superior to the anterior commissure), body (midline structure following the roof of the third ventricle), crura (identified as the caudal bifurcation of the body—ROIs were drawn at the first instance of clear separation in the coronal plane), and fimbria (ROIs were drawn in the first four coronal slices anterior to the inflection point of the crura-to-fimbria curve). For the Cingulum: anterior (Ant-Cing; at the curve of the genu in the axial plane), middle (Mid-Cing; near the middle point between the anterior and posterior ROIs in the coronal plane), and posterior (Post-Cing; at the curve of the splenium in the axial plane) ROIs were drawn. For the MFB: anterior (PFC-MFB; at the level of the prefrontal cortex, PFC, dorsolateral to the corpus callosum genu), middle (NAc-MFB; at the level of the nucleus accumbens, Nac, using the first two coronal slices anterior to the anterior commissure), and posterior (MFB-VTA; just anterior to the ventral tegmental area, VTA) ROIs were drawn.

**Figure 2:**
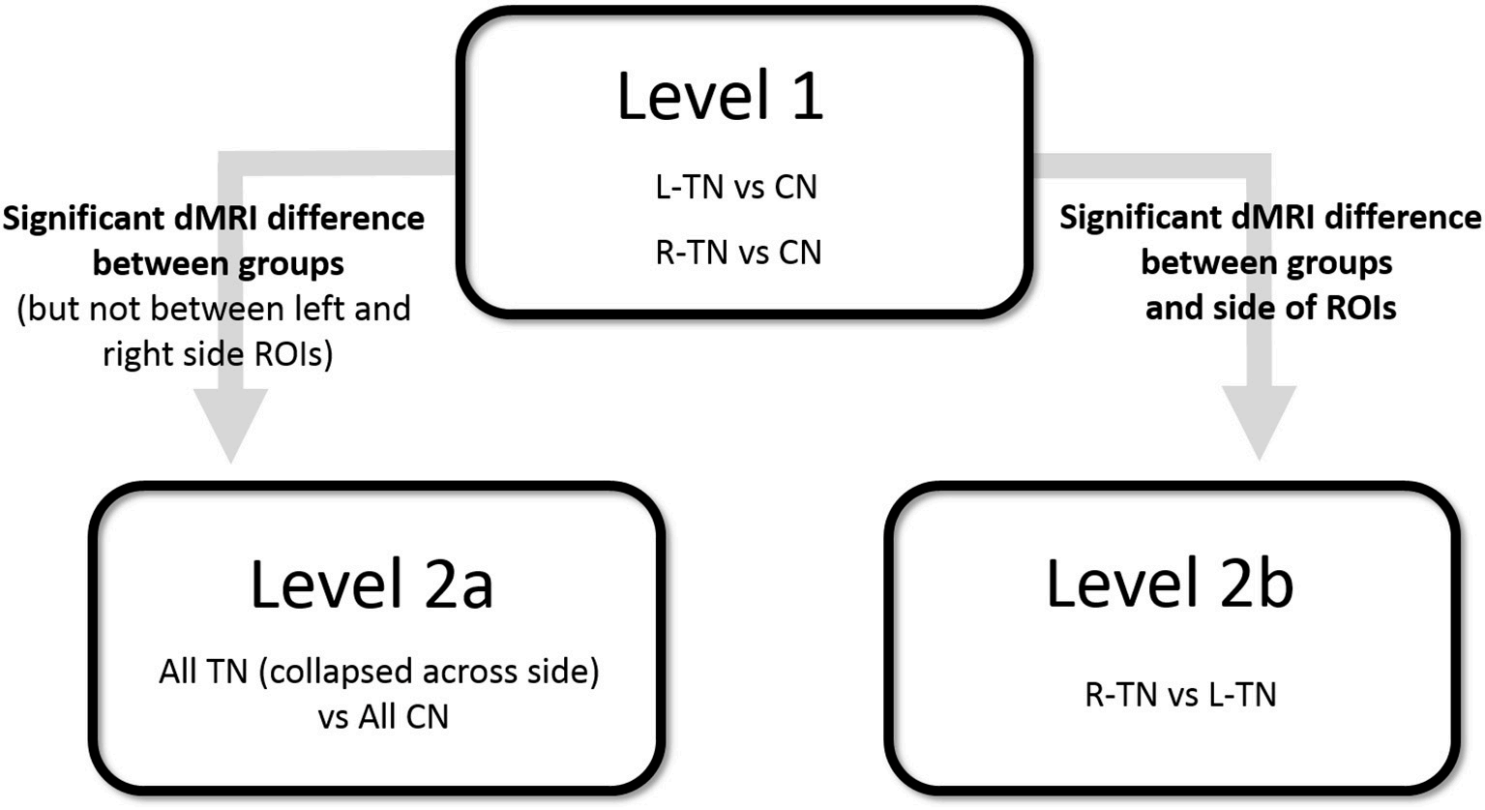
Illustration of statistical design. CN, control subjects; ROI, region of interest; R- and L-TN, trigeminal neuralgia patients with right- and left-sided pain, respectively.

First (i.e., Level 1) and second (i.e., Levels 2a or 2b)-level analyses were performed where appropriate (see Figure 2 for an illustration). A Bonferroni correction for multiple comparisons (i.e., mixed ANOVAs across 6 ROIs × 3 tracts) resulted in an alpha threshold of *p* < 0.002. Statistically significant results from Level 1 led to second-level analyses. Level 1 compared each patient group against their respective control group, i.e., patients with right-sided TN pain (R-TN vs. R-CN) or left-sided pain (L-TN vs. L-CN) vs. controls. Level 2a compared all TN vs. CN when findings from Level 1 indicated that it was appropriate to pool the data from both R-TN and L-TN. Specifically, second-level analysis in Level 2a was used when Level 1 indicated a significant patient vs. control difference in dMRI values but not between the left- and right-sided ROIs in at least one TN group, supporting the notion that the dMRI differences were not specific to one side of the brain. Level 2b directly compared R-TN vs. L-TN when findings from Level 1 indicated that these groups were distinct from both controls as well as each other. Second-level analysis in Level 2b was used when the results from Level 1 indicated an interaction of dMRI metrics and side of ROI or when there were main effects of both dMRI metrics and side of ROI. Repeated measures ANOVAs (2 sides × 4 dMRI metrics) were used to test within- and between-group differences at each pair of ROIs. For Level 1, this resulted in three ANOVAs performed for each of the MFB and cingula, and four ANOVAS for the fornix. Main (sides × dMRI metrics × group) and subordinate interaction terms were considered (i.e., side × dMRI; side × group; dMRI × group). Significant interaction results were followed with appropriate *post-hoc t*-tests. All statistical analyses were performed in SPSS 17.0 (Chicago, IL, USA).

### Gray matter (GM) volumetric ROI analysis

GM volumetric analysis was performed using FreeSurfer software (version 5.3.0; http://surfer.nmr.mgh.harvard.edu/). Briefly, the pipeline includes motion correction, removal of the skull, intensity normalization, transformation to a common Talairach space, and an automated segmentation of cortical and subcortical structures. Further details are discussed elsewhere (e.g., Fischl et al., 2002; Fischl, 2012). For a complete reference of how Freesurfer segments the brain, see Destrieux et al. (2010). Upon completion, each segmented brain was manually inspected, slice by slice, for possible abnormalities and corrected for inaccuracies in the automated tracing algorithm using manually placed control editing points and the auto-reconstruction procedure (McCarthy et al., 2015).

We looked at the volumes of areas immediately innervated by the cingulum (i.e., anterior cingulate, middle cingulate, posterior cingulate, and isthmus), MFB (i.e., nucleus accumbens and ventral diencephalon, which primarily consists of the VTA, substantia nigra, hypothalamus, and mammillary bodies), and fornix (i.e., hippocampus), and which are automatically segmented using Freesurfer. It is worth noting that there is overlapping innervation between many structures, including the MFB and fornix both innervating regions of the ventral diencephalon while the MFB and cingulum both innervate the anterior cingulate cortex. To control for variations in size, all analyses were performed using a ratio of the structure's volume divided by the total intracranial volume of each participant. Each ROI was analyzed using a repeated measures ANOVA (group × side), followed by appropriate *post-hoc* testing (*p* < 0.05). First-stage analysis compared each patient group (those with right-sided, R-TN, or left-sided, L-TN pain) to their respective control group, and this was followed with R-TN vs. L-TN ANOVAs if the first-stage analyses were significant. A Bonferroni correction for multiple comparisons (i.e., mixed ANOVAs across 7 ROIs) resulted in an alpha threshold of *p* < 0.007.

## Results

### Multi-tensor tractography modeling

All 325 tracts were successfully modeled in every subject (see Figure 1 for a typical example at the individual level). However, 19 model tracts were not complete—meaning that they had streamlines which appeared anatomically accurate, but did not allow for the creation of ROIs based on the criteria outlined above. In almost all cases, too few streamlines per voxel precluded ROI drawing. Eight of these were adequately reconstructed following the re-seeding strategy noted above, leaving a final number of 11 incomplete tracts following several re-modeling attempts. These included the following: the fimbria of the fornix was not complete for 3 R-TN patients (2 on right side, 1 on left side), 1 L-TN patient (left side), and 3 healthy controls (right side); one right-sided posterior cingulum model was not complete for a R-TN patient; and 3 left-sided MFB models were not complete at the level of the prefrontal cortex in 3 R-TN patients. ROIs were not drawn in incomplete areas (see above for ROI-drawing criteria), but were successfully drawn for all other sites (i.e., 11 ROIs could not be drawn).

### Model-driven white matter tract ROI analysis

Mean values and standard errors of the mean (SEM) for dMRI metrics extracted from each ROI across groups are displayed in Table 2. The dMRI metrics are fractional anisotropy (FA), and mean (MD), axial (AD), and radial (RD) diffusivities. Values of parametric statistical significance between patient and control groups are indicated in black. For specific significance, see Table 3. Note that the MD, AD, and RD have been scaled up by a factor of 1,000 for interpretive ease.

**Table 2:** Mean dMRI values by ROI.

**Tract**	**ROI**	**dMRI metric** ± **SEM**																															
		**R-CN**								**L-CN**								**R-TN**								**L-TN**							
		**FA**	**±**	**MD**	**±**	**AD**	**±**	**RD**	**±**	**FA**	**±**	**MD**	**±**	**AD**	**±**	**RD**	**±**	**FA**	**±**	**MD**	**±**	**AD**	**±**	**RD**	**±**	**FA**	**±**	**MD**	**±**	**AD**	**±**	**RD**	**±**
Fornix	Column	0.436	0.012	1.482	0.047	2.153	0.051	1.146	0.046	0.435	0.015	1.476	0.059	2.145	0.063	1.141	0.057	0.436	0.017	1.451	0.053	2.128	0.058	1.112	0.053	0.427	0.023	1.482	0.073	2.148	0.077	1.150	0.075
	Body	0.326	0.009	1.487	0.065	2.026	0.089	1.217	0.054	0.324	0.012	1.519	0.084	2.064	0.113	1.247	0.072	0.321	0.009	1.527	0.097	2.084	0.138	1.248	0.078	0.330	0.008	1.645	0.096	2.275	0.140	1.329	0.076
	L_Crus	0.277	0.008	1.749	0.086	2.268	0.112	1.489	0.074	0.256	0.008	1.856	0.117	2.369	0.149	1.600	0.102	0.266	0.010	1.716	0.102	2.206	0.121	1.470	0.093	0.272	0.011	1.737	0.115	2.258	0.147	1.476	0.101
	R_Crus	0.279	0.009	1.632	0.080	2.111	0.097	1.392	0.073	0.275	0.013	1.779	0.105	2.298	0.120	1.519	0.100	0.263	0.009	1.538	0.121	1.967	0.143	1.323	0.110	0.265	0.012	1.627	0.123	2.085	0.143	1.399	0.114
	L_Fimbria	0.526	0.025	0.925	0.034	1.527	0.037	0.624	0.039	0.492	0.030	0.971	0.051	1.543	0.057	0.685	0.054	0.500	0.026	0.933	0.032	1.502	0.045	0.649	0.039	0.466	0.022	0.957	0.027	1.502	0.043	0.684	0.027
	R_Fimbria	0.556	0.022	0.891	0.029	1.517	0.044	0.579	0.026	0.538	0.028	0.956	0.042	1.594	0.038	0.636	0.048	0.532	0.022	0.953	0.061	1.550	0.065	0.654	0.062	0.545	0.021	0.932	0.032	1.562	0.030	0.617	0.035
Cingulum	L_Anterior	0.501	0.017	0.899	0.022	1.459	0.033	0.619	0.023	0.499	0.019	0.897	0.020	1.450	0.030	0.621	0.023	0.477	0.018	0.922	0.016	1.458	0.022	0.654	0.024	0.496	0.018	0.915	0.014	1.474	0.018	0.635	0.022
	R_Anterior	0.466	0.014	0.878	0.017	1.369	0.023	0.632	0.020	0.471	0.017	0.881	0.023	1.390	0.033	0.627	0.024	0.459	0.013	0.897	0.013	1.390	0.019	0.650	0.016	0.455	0.012	0.930	0.014	1.433	0.022	0.678	0.016
	L_Middle	**0.481**	**0.011**	**0.795**	**0.012**	1.265	0.017	**0.560**	**0.014**	**0.483**	**0.013**	**0.789**	**0.018**	1.259	0.025	**0.554**	**0.019**	**0.460**	**0.012**	**0.829**	**0.012**	1.289	0.012	**0.600**	**0.017**	**0.451**	**0.015**	**0.820**	**0.011**	1.256	0.019	**0.602**	**0.014**
	R_Middle	**0.439**	**0.013**	**0.789**	**0.014**	1.193	0.016	**0.587**	**0.016**	**0.453**	**0.014**	**0.777**	**0.021**	1.195	0.028	**0.569**	**0.022**	**0.411**	**0.009**	**0.840**	**0.015**	1.235	0.020	**0.643**	**0.015**	**0.422**	**0.011**	**0.829**	**0.011**	1.231	0.019	**0.628**	**0.012**
	L_Posterior	**0.513**	**0.017**	0.737	0.010	**1.213**	**0.020**	0.499	0.015	**0.520**	**0.022**	0.738	0.010	**1.222**	**0.019**	0.496	0.019	**0.482**	**0.014**	0.791	0.010	**1.263**	**0.015**	0.555	0.014	**0.432**	**0.017**	0.779	0.015	**1.180**	**0.027**	0.578	0.017
	R_Posterior	0.580	0.021	0.719	0.007	1.267	0.031	0.445	0.014	0.532	0.024	0.726	0.007	1.221	0.036	0.478	0.015	0.455	0.017	0.765	0.013	1.193	0.029	0.551	0.013	0.470	0.020	0.763	0.013	1.204	0.036	0.542	0.009
MFB	L_PFC	0.490	0.019	0.792	0.023	1.266	0.026	0.554	0.027	0.513	0.020	0.770	0.017	1.263	0.028	0.523	0.020	0.531	0.026	0.796	0.019	1.328	0.028	0.530	0.025	0.469	0.030	0.774	0.022	1.221	0.045	0.550	0.026
	R_PFC	0.449	0.022	0.789	0.016	1.222	0.034	0.573	0.017	0.474	0.024	0.791	0.019	1.258	0.044	0.558	0.019	0.490	0.019	0.840	0.026	1.348	0.033	0.586	0.029	0.475	0.021	0.801	0.023	1.263	0.034	0.570	0.025
	L_NAc	0.548	0.016	0.802	0.020	1.343	0.022	0.532	0.022	0.588	0.024	0.771	0.015	1.353	0.027	0.480	0.024	0.549	0.018	0.817	0.020	1.383	0.030	0.535	0.022	0.517	0.025	0.853	0.035	1.393	0.048	0.584	0.035
	R_NAc	0.526	0.013	0.808	0.016	1.329	0.027	0.548	0.016	0.542	0.015	0.796	0.016	1.340	0.031	0.524	0.017	0.531	0.015	0.873	0.020	1.458	0.032	0.581	0.020	0.522	0.016	0.865	0.018	1.417	0.029	0.589	0.020
	L_VTA	0.468	0.009	0.861	0.020	1.332	0.036	0.626	0.016	0.454	0.013	0.862	0.030	1.313	0.054	0.636	0.023	0.440	0.010	0.950	0.025	1.426	0.037	0.712	0.023	0.437	0.011	0.918	0.023	1.390	0.037	0.683	0.019
	R_VTA	0.481	0.011	**0.943**	**0.033**	1.465	0.047	**0.682**	**0.029**	0.488	0.014	**0.972**	**0.051**	1.526	0.077	**0.695**	**0.040**	0.459	0.012	**0.968**	**0.029**	1.473	0.037	**0.716**	**0.028**	0.491	0.014	**0.879**	**0.017**	1.392	0.028	**0.622**	**0.017**

*All means dMRI values (FA, MD, AD, RD), and SEM, for each ROI across subject groups. Bolded numbers indicate significant findings described in Table 3. AD, (axial diffusivity); FA, (fractional anisotropy); L-CN, (control group for L-TN); R-CN, (control group for R-TN); MD, (mean diffusivity); RD, (radial diffusivity). MD, AD, and RD were scaled up by 1000x*.

**Table 3:** White matter ROI statistical analyses summary.

**Tract**	**ROI**	**Level 1**								**Level 2a**				**Level 2b**			
		**R-TN vs. CN**				**L-TN vs. CN**				**All TN vs. CN**				**L-TN vs. R-TN**			
		**Interaction**	***F*-value**	***P*-value**	***Post-hoc* tests**	**Interaction**	***F*-value**	***P*-value**	***Post-hoc* tests**	**Interaction**	***F*-value**	***P*-value**	***Post-hoc* tests**	**Interaction**	***F*-value**	***P*-value**	***Post-hoc* tests**
Fornix	Column	NS	–	–	–	NS	–	–	–	–	–	–	–	–	–	–	–
	Body	NS	–	–	–	NS	–	–	–	–	–	–	–	–	–	–	–
	Crura	NS	–	–	–	NS	–	–	–	–	–	–	–	–	–	–	–
	Fimbria	NS	–	–	–	NS	–	–	–	–	–	–	–	–	–	–	–
Cingulum	Anterior	NS	–	–	–	NS	–	–	–	–	–	–	–	–	–	–	–
	Middle	NS, but dxg	*F*_(3, 132)_ = 2.409	0.070	Incr MD/RD	dxg sxd	*F*_(3, 78)_ = 3.137 *F*_(3, 78)_ = 10.23	0.030 <0.0001	Right: Inc MD/RD	dxg	*F*_(3, 189)_ = 5.52	0.001	Bilateral: Dec FA, Inc MD/RD	–	–	–	–
	Posterior	sxdxg	*F*_(3, 129)_ = 9.138	<0.0001	Right: Dec FA, Inc MD/RD Left: Inc MD/RD/AD	dxg	*F*_(3, 78)_ = 9.823	<0.0001	Right: Inc R MD/RD Left: Dec FA, Inc MD/RD	–	–	–	–	sxdxg	*F*_(3, 99)_ = 6.61	<0.0001	L FA: L-TN <R-TN L AD: L-TN > R-TN
MFB	PFC	sxd	*F*_(3, 123)_ = 3.50	0.018	Right: Inc AD	NS	–	–	–	–	–	–	–	–	–	–	–
	NAc	NS	–	–	–	dxg	*F*_(3, 78)_ = 3.50	0.019	Inc MD/RD	NS	*F*_(3, 189)_ = 0.85	0.5	–	–	–	–	–
	VTA	dxg NS, but sxd	*F*_(3, 132)_ = 5.92 *F*_(3, 132)_ = 2.50	0.001 0.063	Left: Dec FA, Inc MD/RD	NS sxdxg sxd	*F*_(3, 78)_ = 0.96 *F*_(3, 78)_ = 16.58	0.42 <0.0001	–	–	–	–	–	sxdxg	*F*_(3, 105)_ = 5.6	0.001	R MD/RD: L-TN <R-TN

*Significant findings following repeated measures ANOVAs (group x dMRI values x side: g x d x s; group x side: g x s; dMRI x group: d x g), and results from post-hoc tests identifying increases (Inc) or decreases (Dec) in dMRI values compared to controls (Levels 1 & 2a) or between patient groups (Level 2b). NS = non-significant for both sxdxg and dxg with reported statistics for sxdxg. Alpha threshold corrected for multiple comparisons is p < 0.002*.

**Figure 3:**
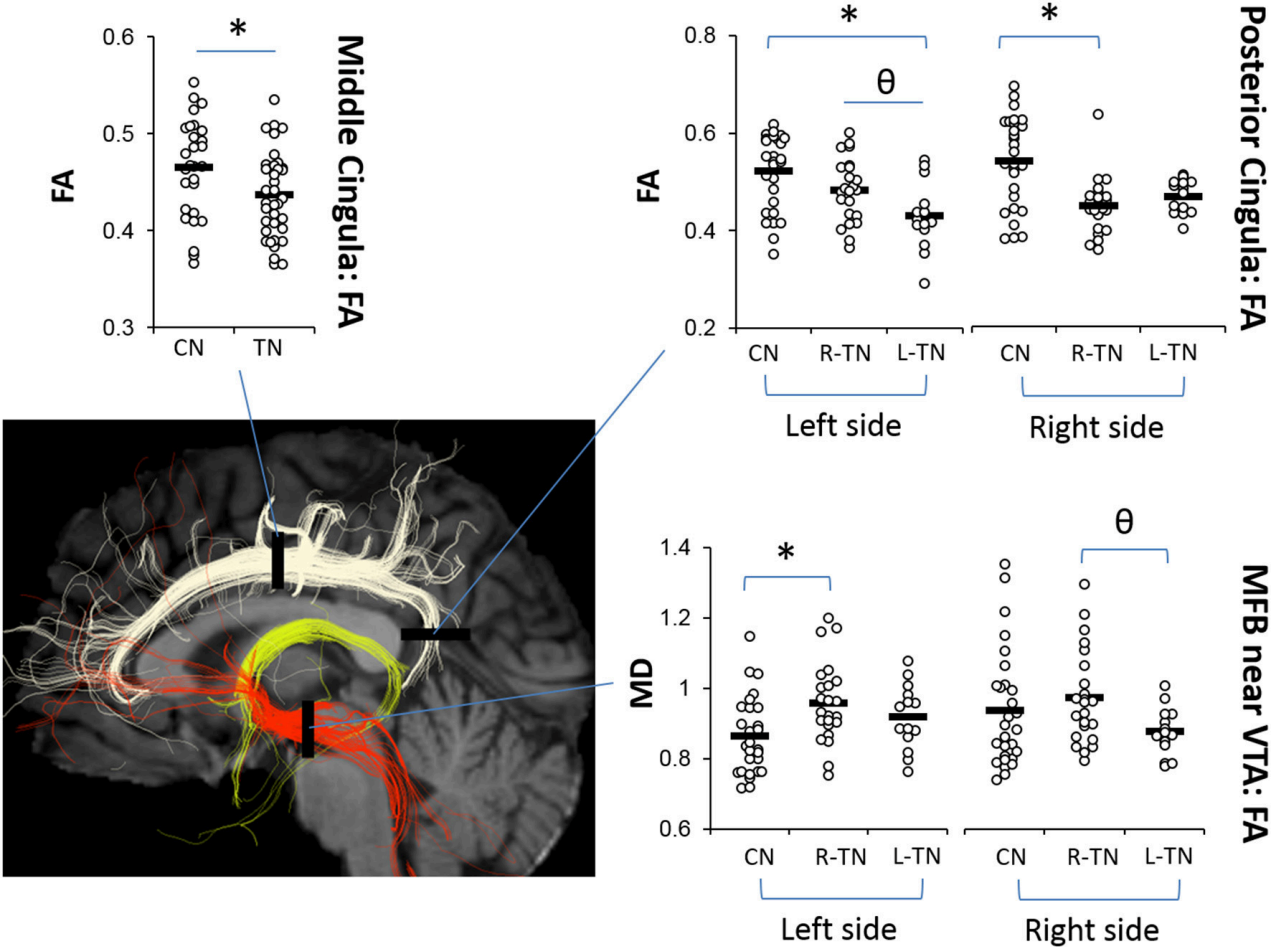
Main dMRI findings in cingula (FA) and MFB-VTA (MD). Scatterplots showing ROIs where fractional anisotropy (FA) for the middle and posterior cingula, and mean diffusivity (MD) for the MFB-VTA, is different from controls (^*^) or between patient groups (θ), indicated by *post-hoc* tests (*p* < 0.05) following repeated measures ANOVA. MD was scaled up by 1000x. Corresponding changes in other dMRI metrics for these regions are indicated in Table 3.

The results from significant statistical analyses can be found in Table 3. The main FA findings are illustrated using scatterplots (Weissgerber et al., 2015) in Figure 3, and all correspond to changes in MD and also changes in either AD, RD, or both. Briefly, no significance (NS) was seen for any forniceal ROIs. For the cingula, both the middle and posterior ROIs were significantly different. The middle ROI showed a difference for L-TN vs. L-CN for the dMRI × group interaction (dxg: *p* = 0.03), but not for the R-TN vs. R-CN (dxg: *p* = 0.07), while comparing all TN patients vs. controls revealed a significant bilateral decrease in FA (see Figure 3) and increases in MD and RD (*p* = 0.001). The posterior ROI showed interactions between each patient group and their respective control groups which was significant for the side of ROI. Level 2b analysis confirmed this R-TN vs. L-TN difference showing particularly that FA and AD were significantly different between these groups on the left side of the brain (*p* < 0.0001). Finally, the MFB showed differences at the level of the VTA in L-TN and R-TN patients compared to their control groups (*p* = 0.001; Figure 3)—revealing that MD and RD are lower in the right-side of L-TN, when compared to R-TN, patients. See Supplementary Table 1 for results from all non-significant findings.

### Gray matter ROI analysis

**Figure 4:**
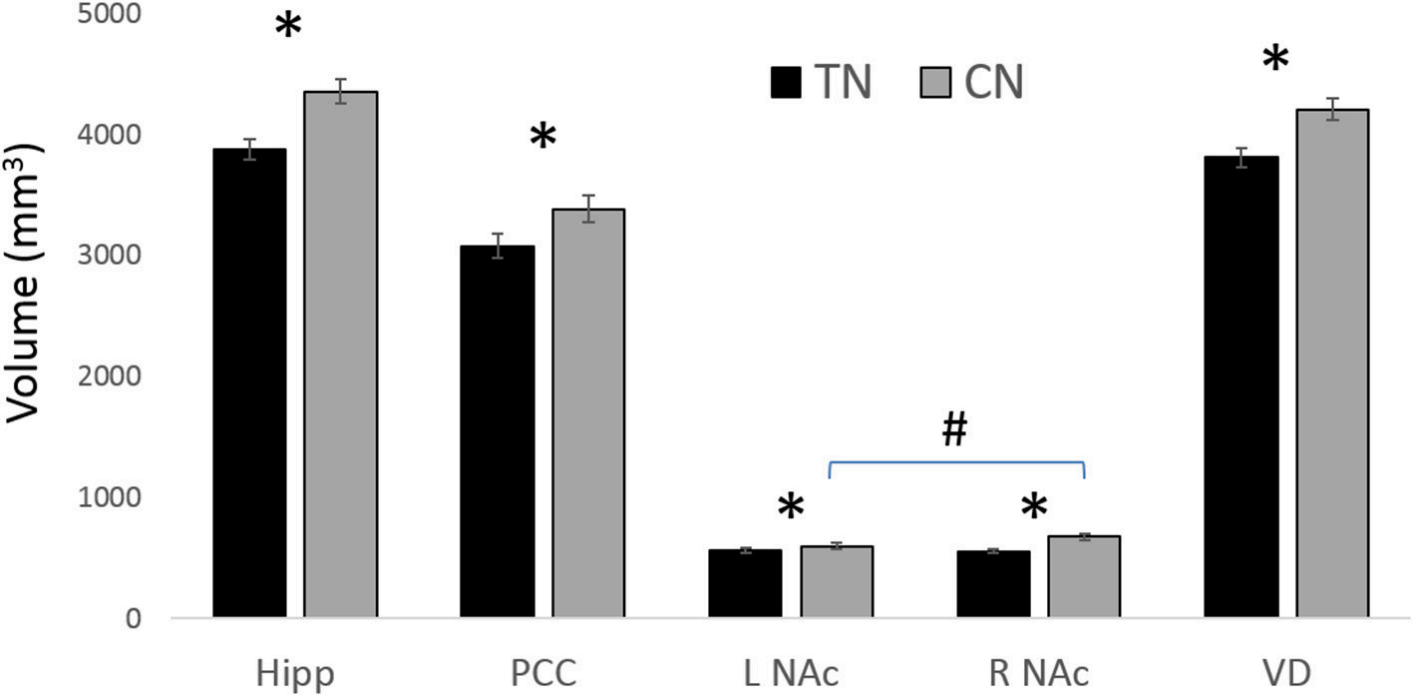
Main GM findings. The main results showing gray matter (GM) decreases in patients with TN compared to healthy controls (CN). ^*^ = significant between groups; # L NAc is smaller than R NAc in CN; all results are significant at *p* < 0.05 following ANOVA with corrections for multiple comparisons. Hipp, (Hippocampus); NAc, (nucleus accumbens); PCC, (posterior cingulate cortex); VD, (ventral diencephalon). See Table 4 for corresponding statistics.

All statistically significant results are noted in Table 4 and illustrated in Figure 4. In brief, we investigated the volumes of all four major subregions of the cingulate cortex, the NAc, VD, and the hippocampus. We noted significant bilateral volumetric decreases in the posterior cingulate, NAc, VD, and hippocampus in both TN groups compared to healthy controls. Alternately, no differences were noted in the rostral ACC, middle ACC, or isthmus.

**Table 4:** Gray matter ROI statistical analyses summary.

**Associated Tract**	**ROI**	**R-TN vs. CN**				**L-TN vs. CN**			
		**Interaction**	***F*-value**	***P*-value**	***Post-hoc* tests**	**Interaction**	***F*-value**	***P*-value**	***Post-hoc* tests**
Fornix	Hippocampus	g sxg	19.33 2.0	<0.0001 0.16	TN < CN bilaterally	g sxg	12.76 12.72	0.001 0.003	TN < CN bilaterally
Cingulate	Anterior	NS (after MC)	–	–	–	NS (after MC)	–	–	–
	Middle	NS (after MC)	–	–	–	NS (after MC)	–	–	–
	Posterior	g sxg	16.05 1.43	0.0002 0.24	TN < CN bilaterally	g sxg (NS)	15.04 0.165	0.001 0.7	TN < CN bilaterally
	Isthmus	NS (after MC)	–	–	–	NS	–	–	–
MFB	NAc	g sxg	17.09 11.41	0.0001 0.002	TN < CN bilaterally, but only CN show L-NAc < R-NAc	g sxg	13.27 8.56	0.001 0.007	TN < CN bilaterally, but only CN show L-NAc < R-NAc
	Ventral diencephalon (including midbrain and hypothalamus)	g sxg	22.67 3.62	<0.0001 0.06	TN < CN bilaterally	g sxg	13.73 2.5	0.001 0.13	TN < CN bilaterally

*Significant findings for repeated measures ANOVAs (group: g; group x side: g x s), and results from post-hoc tests identifying changes in gray matter volumes (mm^3^) in TN patients vs. healthy controls (CN). Alpha threshold corrected for multiple comparisons (MC) is p < 0.007*.

## Discussion

This is the first study to investigate the potential relationship between the structure of major affective brain circuits (Figure 1) in those with TN (Table 1). In line with our first hypothesis, all 325 tracts across TN patients and healthy controls were successfully modeled, with only 11/1170 unusable ROIs (due to partially incomplete tracts). Of the three affect-related tracts, the cingula and MFB revealed alterations in those with TN (Tables 2, 3). Alterations in dMRI metrics of the middle and posterior cingulum, and the MFB near the VTA, along with decreased gray matter (GM) volumes in the posterior cingulate (PCC), nucleus accumbens (NAc), and ventral diencephalon (VD), further suggest that chronic neuropathic pain associated with TN is related to changes in the microstructure of these circuits (Figures 3, 4, Table 4). Unexpectedly, no clear differences were noted for the anterior cingulum, PFC-MFB, or NAc-MFB white matter ROIs (Tables 2, 3) or for the rostral ACC, middle ACC, or isthmus GM ROIs (Table 4). As anticipated, the fornix—along the four major subdivisions (Chen et al., 2015) studied here—did not demonstrate any alterations, further suggesting that affective circuitry is not indiscriminately altered in those with TN. Finally, decreased bilateral hippocampal volumes in TN are consistent with changes seen in other pain- and stress-related disorders, however we were unable to compare most of the associated fimbria in this region due to limitations of the tractography modeling.

Taken together, these results support the notion of selectively altered affective circuits in patients with TN which may be related to their experience of negative affect and the comorbidity of mood and anxiety disorders seen in this population. Moreover, these findings should be considered in the design of future studies aimed at elucidating how changes in white and gray matter structures—which are neuroanatomically remote from the trigeminal nerve—can help to further characterize patients with TN.

### Affective circuit disruptions in TN: mid-cingulum, post-cingulum and PCC, and MFB-VTA and VD

The middle and posterior sections of the cingulate cortex have been associated with the processing of the affective, sensory, and perceptual dimensions of pain as well as cognitive-attentional control (Shackman et al., 2011; Leech and Sharp, 2014). The cingulum is the major white matter tract running along the cingulate cortices, providing broad direct connectivity between the prefrontal and posterior cingulate cortices, and the midcingulum also sends local projections upward to the sensorimotor, frontal, and parietal cortices (Jones et al., 2013; Heilbronner and Haber, 2014). Here, we replicated prior findings from our group (DeSouza et al., 2014) showing alterations in dMRI metrics for TN patients compared to healthy controls, i.e., decreased FA and increased MD and RD, in the bilateral midcingula (Figure 3, Table 3). These were found using the multi-tensor tractography model-driven ROI approach, while prior findings were uncovered using a whole-brain voxel-wise approach, i.e., FSL's TBSS. The identified role of the midcingulate region as a hub involved in the affective dimension of pain has been well-established by the abundant translational evidence (Hayes and Northoff, 2012), although how precisely it is involved in the integration of processing related to affect, salience, and pain requires further study (Shackman et al., 2011).

We also extended upon prior findings by showing changes in the posterior cingulum and PCC which were significant across all groups, i.e., bilateral decreases in PCC volume along with decreased FA on the patient's affected side, and increased MD and RD bilaterally in both R-TN and L-TN patients (Tables 3, 4). This is consistent with nearby changes to the posterior corona radiata, posterior superior longitudinal fasciculus, and posterior corpus callosum identified previously (DeSouza et al., 2014) as these nearby regions share overlapping innervation. In fact, given the smoothing and registration steps involved in whole-brain voxel-wise analyses used previously, and the proximity of these other tracts to the cingulum, it is possible that some of the nearby changes identified previously are actually alterations of the cingulum (Bach et al., 2014)—however, we did not test this explicitly here as it would require the independent reconstruction of these more complex highly-crossing tracts. TN-related alterations of the posterior cingulum—which connects the nearby posterior cingulate, precuneus, retrosplenial/isthmus, and parietal cortices to frontal, temporal, and subcortical sites (Jones et al., 2013; Heilbronner and Haber, 2014)—was anticipated, as the posterior cingulate-precuneus has been identified as a functionally relevant area in previous chronic pain neuroimaging studies. In particular, the posterior cingulate is a key structure of the default mode network, and altered structural-functional connectivity within this network and sensorimotor regions has been identified in patients with fibromyalgia (Cifre et al., 2012; Flodin et al., 2014), temporomandibular disorder (Weissman-Fogel et al., 2011; Kucyi et al., 2014), and chronic back pain (Kregel et al., 2015).

The current inclusion of an L-TN group, unlike prior studies which included only patients with R-TN, allowed for the investigation of potential differences between these patient groups—this is also why our first stage of analysis considered each R-TN and L-TN group separately. Identification of unilateral differences in FA, however, was unexpected. This finding suggests that those suffering from right-sided TN pain may show greater alterations in their right-sided posterior cingulum, and vice versa for L-TN patients (although right-side FA was also significantly decreased in L-TN patients), when compared to healthy controls as well as when compared between patient groups (Figure 3). Although unexpected, a few reports are in line with such unilateral findings, as patients with fibromyalgia show altered processing in posterior cingulate cortex and, ipsilateral to a painful stimulus, secondary somatosensory cortex (Gracely, 2004). Also, those with complex regional pain syndrome show increased activation of the posterior cingulate during stimulation of their symptomatic hand (Freund et al., 2010). Further study is required to fully understand the significance of this unilateral difference, especially since only bilateral and not unilateral decreases in PCC volume were noted here.

Interestingly, our results suggest that the left-sided MFB, just anterior to the VTA region, may be different in patients compared to controls. We noted dMRI changes (i.e., decreased FA, increased MD and RD) in R-TN patients in the left MFB-VTA when compared to healthy controls (Figure 3, Table 3; however, the L-TN group showed no dMRI changes, which may be due to the latter group's smaller size). These results are also consistent with the decreased volumes of the VD (consisting mainly of the VTA, substantia nigra, hypothalamus, and mammillary bodies) seen here (Figure 4), although this decrease was bilateral in both R-TN and L-TN groups. Although few studies have considered laterality differences for the MFB or VTA, one recent study showed the left MFB FA correlated with the ability to experience pleasure (Bracht et al., 2015). The MFB connects the VTA, NAc, and PFC, and has been successfully reconstructed by others using tractographic models (Coenen et al., 2012b; Hana et al., 2015). It is strongly implicated in the processing of appetitive and aversive stimuli (Hayes et al., 2014), and the VTA is important in human pain processing (Fairhurst et al., 2007; Hayes and Northoff, 2012). Animal studies have identified a host of neurochemical factors involved in regulating the nociceptive response within the VTA, including a complex involvement of orexin, opioids, and dopamine (Yazdi-Ravandi et al., 2014; Hipolito et al., 2015). Increased VTA microglial activation is associated with chronic neuropathic pain (Taylor et al., 2015), and is one possible explanation for the altered dMRI metrics in the present study, as increased microglial activation reflects local inflammation which may lead to disruptions in water diffusivity, decreased FA, and increased MD and RD (Alexander et al., 2007; Po et al., 2012).

Interestingly, previous animal studies have described asymmetrical structure and function within the mesocorticolimbic system with a general bias for left-sided aversive, and right-sided appetitive, processing found in some studies (Rosen et al., 1984; Besson and Louilot, 1995). This makes our second-level finding of lower MD and RD values in L-TN compared with R-TN patients intriguing—however, at this point, the relevance of this finding is unclear and is not further supported by the GM findings. Taken together, these results suggest that the alterations in the left-sided MFB-VTA tract and bilateral decreases in the VD seen in our study may reflect altered aversion-related processing, broadly consistent with the experience of chronic neuropathic pain and persistent negative affect.

### Disruptions are not evenly distributed: ant-cingulum, PFC-MFB, NAc-MFB, and fornix

The present study included the fornix as a tract involved in affective processing, but which has not been consistently identified as a structure impacted in chronic neuropathic pain. In fact, only one study links chronic pain to changes in the fornix, finding higher FA in the whole fornix of patients with irritable bowel syndrome (Chen et al., 2011). However, these findings should be considered cautiously as there were only 10 patients analyzed compared to controls (*p* = 0.014) using whole-brain voxel-wise analysis (Bach et al., 2014; and see discussion above). Consistent with our hypothesis, we found no forniceal differences in the present study between TN patients and controls—across ROIs which were chosen based on previously identified anatomical subdivisions (i.e., column, body, crura, and fimbria) (Chen et al., 2015). However, a major GM target of the fornix, the hippocampus, did show decreased volumes bilaterally in TN patients. This is consistent with prior evidence showing decreased hippocampal volumes in response to chronic pain and stress (although see Smallwood et al., 2013 for evidence that the right hippocampus might actually be larger in some cases) and may also correspond to decreases in anterior fimbria FA values—although tractographic modeling prevented us from investigating this in the present study (see limitations below for further discussion).

Contrary to our original hypotheses, however, we found no changes within the anterior cingulum (Ant-Cing), or along the MFB at the levels of the PFC or NAc/ventral striatum (Supplementary Table 1). Projections from both the Ant-Cing and PFC-MFB enter the medial prefrontal cortex, a region involved in the processing and contextualization of healthy affective responses (Roy et al., 2012; Hayes et al., 2014), and which has shown decreased gray matter volume and altered FA in other studies of chronic neuropathic pain (Moayedi et al., 2012; Ivo et al., 2013; Lin, 2014). Nonetheless, our current findings are in line with prior studies in TN showing white matter changes primarily in middle and posterior tracts (Gustin et al., 2012; Moayedi et al., 2012; DeSouza et al., 2014). Importantly, the absence of findings here does not preclude the involvement of the prefrontal cortex in TN, as other studies have shown it to predict painful responsivity and exposure to childhood stressors despite not showing direct intraregional functional or structural changes (Hayes et al., 2013; Duncan et al., 2015). The splayed nature of the PFC-MFB tract also resulted in a greater variability in ROI placement compared to all other ROIs, which were relatively anatomically confined, and it is possible that this variability may have prevented the identification of clear changes in this region.

Perhaps the most unexpected negative finding was the absence of changes in NAc-MFB microstructure, despite the fact that the MFB was well-modeled at this level in all subjects (Figure 1). This was not expected given prior findings in both rats (Chang et al., 2014) and humans (Baliki et al., 2010; Hashmi et al., 2013) of NAc functional changes and the role of PFC-NAc connectivity in subjects with chronic neuropathic pain (Baliki et al., 2012; Lee et al., 2015). Alternately, the finding of decreased NAc volumes (Figure 4, Table 4) in TN patients is consistent with the role of this structure in pain and affective processing and is in agreement with prior pain studies (Geha et al., 2008; Gustin et al., 2011). Although our NAc-MFB ROIs were selected using clear anatomical landmarks (Lucas-Neto et al., 2014), it remains the case that fibers from the MFB are intertwined with the fibers of the anterior limb of the internal capsule at this level (Coenen et al., 2012a,b; Hana et al., 2015), and so selected voxels will likely contain many non-MFB fibers in this region compared to the MFB-VTA.

### Limitations and future directions

This study investigated the hypothesis that chronic pain and associated negative affect associated with TN would correspond to changes in major limbic circuits, as seen in altered white and gray matter. The patients were candidates for neurosurgery and, as such, were treatment-resistant and reported the most intense pain imaginable on standard scales. As is typical in these patients, all reported the highest levels of subjective pain (a 9 or 10 on a 10-point scale), and many reported subjective changes in anxiety or mood over time. Unfortunately, there was not enough variability to look for correlations related to subjective pain reports, and no standard measures of mood or anxiety were taken during clinical visits. We intend to include such measures in future.

Psychosocial comorbidities are known to play a significant role in the risk of developing, and progression of, chronic neuropathic pain syndromes (Turk et al., 2010; Simons et al., 2013). The TN patients in the present study were screened as potential surgical candidates and were included here because there were no obvious psychiatric or psychological disorders present. It should be noted, however, that this is not assessed through formal psychiatric screening, but by standard neurological and neurosurgical clinical assessments. Nearly all patients were on some form of medication (Table 1)—typically antiepileptics whose effects on dMRI metrics have not been studied. Although the effects of these drugs have not been well-studied in this regard, there is evidence that the long-term use of antiepileptics (such as carbamazepine, the main pharmacological treatment used in our sample) in those with temporal lobe epilepsy results in alterations in the hippocampus, posterior cingulate, and thalamus (Haneef et al., 2015). In particular, the authors found reduced betweenness centrality (a measure of connectivity, or “hubness,” used in graph theory) in the hippocampus and increased betweenness centrality in the cingulate—and particularly in the posterior cingulate. This raises the possibility that our findings, particularly for the posterior cingulate, may be related in part to the long-term use of these compounds. Further research is needed to disentangle these effects.

The three tracts were chosen for their role as major affective pathways, as well as their relatively low-variability across subjects. Nonetheless, a primary limitation of the current approach is the increased variability in some subregions (e.g., PFC-MFB) over others as well as the inability to explore all anatomical subregions in detail. For instance, although we saw no fornix changes in the present analysis, there could be subtler changes in the finer projections of the pre- and post-commissural fornix, as these project to highly relevant affective regions such as the accumbens and hypothalamus, or in the anterior portion of the fimbria. Indeed, although white and gray matter changes do not necessarily occur in unison, the extent to which the hippocampal volumes are decreased here raises the possibility that the fimbria may also be altered. The anterior portion of the fimbria is especially interesting given that many human and animal studies related to chronic stress or pain have shown appreciable changes in hippocampal volumes (Bremner, 2006; Smallwood et al., 2013; Vachon-presseau et al., 2013; Vachon-Presseau et al., 2016).

## Conclusion

This is the first study to use an *a priori* investigation of the impact of TN on affective circuits, finding white matter alterations in the middle and posterior cingulum and near the MFB-VTA, and corresponding gray matter changes in the PCC and VD as well as decreased NAc volumes. Based on these promising findings, we are currently undertaking studies to determine if these abnormalities are linked directly with other functional brain differences and affective behavioral measures. We will also look at whether post-surgical outcomes of patients who received neurosurgical interventions, have reversals of such alterations following successful surgeries. Further studies should also look at whether these findings can be used to help more accurately tailor clinical and surgical approaches to individuals, as there are numerous surgical approaches available for which it is currently challenging to predict favorable outcomes.

## Author contributions

DH, DC, BB, MW, JZ, AL, and MH contributed to the experimental design and analysis. DC and MH collected the data. DH and MH wrote the paper.

### Conflict of interest statement

The authors declare that the research was conducted in the absence of any commercial or financial relationships that could be construed as a potential conflict of interest.

## Abbreviations

AD: axial diffusivity
dMRI: diffusion magnetic resonance imaging
FA: fractional anisotropy
GM: gray matter
Hipp: hippocampus
MD: mean diffusivity
MFB: medial forebrain bundle
NAc: nucleus accumbens
PCC: posterior cingulate
PFC: prefrontal cortex
RD: radial diffusivity
ROI: region of interest
TN: trigeminal neuralgia
VD: ventral diencephalon
VTA: ventral tegmental area
WM: white matter.
