# Supplementary material for: Affective Circuitry Alterations in Patients with Trigeminal Neuralgia
Source: Front Neuroanat. 2017 Sep 5;11:73. doi: 10.3389/fnana.2017.00073 (PMC5591854; doi:10.3389/fnana.2017.00073)
Supplement: Supplementary file 1 [file Table1.docx]

**Supplementary Table 1: All model 1 statistical analyses summary by ROI (dMRI metric x group)**

| **Tract** | **ROI** | **R-TN v CN** | | | | **L-TN v CN** | | | |
| --- | --- | --- | --- | --- | --- | --- | --- | --- | --- |
|  |  | **Interaction** | **F value** | **P value** | **Posthoc tests** | **Interaction** | **F value** | **P value** | **Posthoc tests** |
| **Fornix** | **Column** | NS | F(3,132)=0.1 | 0.96 | - | NS | F(3,78)=0.07 | 0.98 | - |
|  | **Body** | NS | F(3,132)=0.21 | 0.89 | - | NS | F(3,78)=0.14 | 0.94 | - |
|  | **Crura** | NS | F(3,132)=1.99 | 0.90 | - | NS | F(3,78)=0.04 | 0.99 | - |
|  | **Fimbria** | NS | F(3,117)=0.09 | 0.97 | - | NS | F(3,63)=0.79 | 0.504 | - |
|  |  |  |  |  |  |  |  |  |  |
| **Cingulum** | **Anterior** | NS | F(3,132)=0.52 | 0.67 | - | NS | F(3,78)=0.18 | 0.91 | - |
|  | **Middle** | NS, but dxg | F(3,132)=2.409 | 0.070 | Incr MD/RD | dxg sxd | F(3,78)=3.137 F(3,78)=10.23 | 0.030 <0.0001 | Right: Inc MD/RD |
|  | **Posterior** | sxdxg | F(3,129)=9.138 | <0.0001 | Right: Dec FA, Inc MD/RD Left: Inc MD/RD/AD | dxg | F(3,78)=9.823 | <0.0001 | Right: Inc R MD/RD Left: Dec FA, Inc MD/RD |
|  |  |  |  |  |  |  |  |  |  |
| **MFB** | **PFC** | dxg  sxd | sxd | F(3,123)=3.50 | 0.018 | Right: Inc AD | NS | F(3,78)=0.90 | 0.44 |
|  | **NAc** | NS | NS | F(3,132)=0.009 | 0.99 | - | dxg | F(3,78)=3.50 | 0.019 |
|  | **VTA** | dxg  sxd | dxg NS, but sxd | F(3,132)=5.92 F(3,132)=2.50 | 0.001 0.063 | Left: Dec FA, Inc MD/RD | NS sxdxg sxd | F(3,78)=0.96 F(3,78)=16.58 | 0.42 <0.0001 |
